# Pediatric ependymoma: an overview of a complex disease

**DOI:** 10.1007/s00381-021-05207-7

**Published:** 2021-05-18

**Authors:** Stephanie Theresa Jünger, Beate Timmermann, Torsten Pietsch

**Affiliations:** 1 Department of Neuropathology, DGNN Brain Tumor Reference Centre, University of Bonn Medical Centre, Bonn, Germany; 2 Centre for Neurosurgery, Department of General Neurosurgery, Faculty of Medicine and University Hospital Cologne, University of Cologne, Cologne, Germany; 3 Department of Particle Therapy, University Hospital Essen, West German Proton Therapy Centre Essen (WPE), West German Cancer Center (WTZ), German Cancer Consortium (DKTK), Essen, Germany

**Keywords:** Ependymoma, Pediatric, Genetics, Chemotherapy, Radiation, Neuropathology

## Abstract

Pediatric ependymomas comprise biologically distinct tumor entities with different (epi)genetics, age distribution and localization, as well as a different prognosis. Regarding risk stratification within these biologically defined entities, histopathological features still seem to be relevant. The mainstay of treatment is gross total resection (GTR) if possible, achieved with intraoperative monitoring and neuronavigation—and if necessary second surgery—followed by adjuvant radiation therapy. However, there is growing evidence that some ependymal tumors may be cured by surgery alone, while others relapse despite adjuvant treatment. To date, the role of chemotherapy is not clear. Current therapy achieves reasonable survival rates for the majority of ependymoma patients. The next challenge is to go beyond initial tumor control and use risk-adapted therapy to reduce secondary effect and therapy-induced morbidity for low-risk patients and to intensify treatment for high-risk patients. With identification of specific alterations, targeted therapy may represent an option for individualized treatment modalities in the future.

## Introduction

Ependymomas represent the third most common malignant intracranial neoplasm in children. Pediatric ependymomas can occur in all compartments of the CNS; the most frequent location is the posterior fossa followed by supratentorial sites, while spinal tumors are rather rare [1, 2]. A slight overall male predominance is reported [1–3]. Based on their histological resemblance, ependymomas across all locations were historically considered as one entity but assigned different tumor grades. Assuming that histopathological indicators of anaplasia, namely mitotic activity, vascular proliferation, and necrosis, were adequate predictors for outcome independent of patient age and tumor location, WHO grading was used for risk stratification and hence for treatment decisions. However, the utility of grading for risk stratification has remained controversial, especially because of its lack of reproducibility due to inter-observer variability [4, 5].

Nevertheless, to date, histological diagnosis remains important since it can be performed immediately and almost everywhere. However, genetic and epigenetic studies in ependymomas have led to the identification of biologically distinct subtypes, suggesting more adequate means for risk stratification [6–14].

Taylor et al. were able to show that tumors of different locations in the CNS represent distinct tumor entities derived from spatially restricted progenitor cell populations of radial glia cells, indicating that spatial heterogeneity may be associated with distinct tumor biology [13]. These findings indicate that tumors in different locations constitute genetically and biologically distinct diseases despite their histopathological resemblance. Pajtler et al. presented an epigenetic classification of ependymomas which divides supra-, infratentorial and spinal ependymomas into three different, partly age-related, and prognostically relevant groups each: spinal subependymoma (SP-SE), spinal myxopapillary ependymoma (SP-MPE), spinal ependymoma (SP-EPN), posterior fossa subependymoma (PF-SE), posterior fossa ependymoma-A (PF-EPN-A), posterior fossa ependymoma-B (PF-EPN-B), supratentorial subependymoma (ST-SE), supratentorial ependymoma YAP1-fused (ST-EPN-YAP), and supratentorial ependymoma RELA-fused (ST-EPN-RELA) [11].

In this review, the subdivision is partly orientated on the classification by Pajtler et al. However, pediatric ependymoma is distinct from adult tumors, as indicated in the section below; therefore, we aimed to focus on ependymomas occurring in childhood. Current treatment modalities will be discussed separately, since most trials published to date have used histopathological grade, age, and extent of resection for stratification rather than localization or epigenetically defined subgroups. Information regarding studies identifying prognostic parameters and results from prospective clinical trials provided in the text are summarized in Tables 1 and 2.

**Table 1:** Identification of prognostic parameters in pediatric ependymoma (review of the literature)

Study	Location	No. of patients (n), pediatric (p), adult (a), mixed (m)	Results
Scheil et al. (2001)	ST, PF, SP	*n*=22; *m*	Chromosome 1q gain only present in pediatric EP, *N-MYC* amplification present in adult spinal EP
Dyer et al. (2002)	ST, PF, SP	*n*=53; *p*	Characterization of 3 genomic EP types with possible prognostic relevance: numerical, structural associated with chromosome 1q gain and an inferior prognosis, balanced predominant in infancy
Taylor et al. (2005)	ST, PF, SP	*n*=103; *m*	Spatially defined radial glia cells are the cell of origin of ependymomas, with different genetic characteristics despite histological resemblance
Benesch et al. (2010)	SP	*n*=29; *p*	Extent of resection was the strongest predictor of outcome mainly PFS, the role of adjuvant radio-chemotherapy still needs to be defined, due to risk of recurrence close surveillance is warranted
Korshunov et al. (2010)	ST, PF, SP	*n*=122; *m*	EPN with chromosome 1q gain and homozygous *CDKN2A* deletion showed an inferior prognosis
Witt et al. (2011)	ST, PF, SP	*n*=177; *m*	PF-EPN may be divided into two groups A and B with A showing a lateral growth pattern, cerebellar invasion, and a poor prognosis event-free and overall survival, while B may be predominant in older patients, representing with a less invasive/aggressive growth and a favorable prognosis
Kilday et al. (2012)	PF	*n*=48; *p*	Pediatric PF-EPN with chromosome 1q gain and incomplete resection showed an inferior prognosis
Godfraind et al. (2012)	ST, PF	*n*=146; *p*	Residual tumor, gain of chromosome 1q, and high mitotic activity were predictors for an inferior prognosis in pediatric PF-EPN, alike were brain invasion and homozygous *CDKN2A* deletion in ST-EPN
Parker et al. (2014)	ST	*n*=77; *m*	Characterization of ST EPN with *RELA* and *YAP* fusions
Pietsch et al. (2014)	ST	*n*=17; *p*	Characterization of ST EPN with *RELA* fusion in pediatric patients
Mack et al. (2014)	PF	*n*=47; *m*	PF-EPN may be divided into two epigenetically defined groups according to CpG island methylator phenotype, with the methylated one depicting a poor prognosis PF-EPN-A
Pajtler et al. (2015)	ST, PF, SP	*n*=498; *m*	Methylation-based classification, identifying 9 subgroups stratified according to location: SP-SE, SP-MPE, SP-EPN, PF-SE, PF-EPN-A, PF-EPN-B, ST-SE, ST-EPN-YAP, ST-EPN-RELA; ST-EPN-RELA and PF-EPN-A with poor and ST-EPN-YAP, PF-EPN-B, and SE with favorable prognosis
Bayliss et al. (2016)	ST, PF, SP	*n*=20; *m*	Epigenetically driven H3K27me3 loss may be detected by immunohistochemistry to identify pediatric PF-EPN-A, which showed an inferior prognosis
Ramaswamy et al. (2016)	PF	*n*=820; *m*	PF-EPN-A displayed a dismal prognosis compared to PF-EPN-B, which may be cured by surgery alone
Panwalkar et al. (2017)	PF	*n*=460; *m*	H3K27me3 immunohistochemistry reliably identified PF-EPN-A
Cavalli et al. (2018)	PF	*n*=212; *m*	Characterization of 5 PF-EPN-B subgroups with individual methylation profiles associated with an overall favorable prognosis but possible late relapses across all groups; extent of resection represents the strongest prognostic parameter across all PF-EPN-B groups
Fukuoka et al. (2018)	ST, PF	*n*=107; *m*	Characterization of ST-EPN without *RELA* and *YAP* fusions but alternative fusion-transcripts; validation of poor prognosis in PF-EPN-A
Pajtler et al. (2018)	PF	*n*=675; *m*	Methylation-based subclassification of EPN-PF-A with distinct prognosis: PFA-1c enriched by chromosome 1q gain showed a poor prognosis, while PFA-2c with OTX2 expression displayed a favorable prognosis, and PFA-1f may harbor H3K27me3 mutations
Witt et al. (2018)	ST, PF, SP	*n*=122; *a*	PF-EPN-A are almost absent in adult patients, EPN in adulthood showed a favorable prognosis, molecular classification may provide a more precise classification beyond histology
Andreiuolo et al. (2019)	ST	*n*=15; *p*	Characterization of pediatric ST-EPN with *YAP* fusion with a favorable prognosis
Benesch et al. (2019)	ST, PF	*n*=10 (primarily metastatic); *p*	Primarily metastatic EPN were rare in pediatric patients, ST-EPN-RELA with radio-chemotherapy showed a better prognosis compared to PF-EPN- A
Ghasemi et al. (2019)	SP	*n*=13; *m*	*N-MYC* amplification occurred in spinal EP II and III and depicts a negative predictor of outcome
Jünger et al. (2019)	PF	*n*=134; *p*	Integrated risk stratification for pediatric PF-EPN with chromosome 1q gain, incomplete resection, and high mitotic activity as negative prognostic parameters
Swanson et al. (2019)	SP	*n*=4; *m*	*N-MYC* amplification occurred in spinal EP III and was an indicator for poor PFS and OS disregarding administered therapy
Jünger et al. (2020a)	ST	*n*=54; *p*	Identification of *CDKN2A* deletion as adverse prognostic factor in pediatric ST-EPN-RELA
Jünger et al. (2020b)	ST, PF	*n*=28; *p* (<3 years)	Infant PF-EPN-A showed completely balanced genomes, *CDKN2A* deletion were absent in very young children with ST-EPN
Neumann et al. (2020)	ST, PF, SP	*n*=48; *m*	Integrated diagnostic methods in EP are warranted since histology alone may be insufficient; ependymoma with clear cell and papillary morphology may represent distinct tumors with respect to morphology, location, and methylation profile, while tanycytic ependymoma may not do so
Pagès et al. (2020)	ST	*n*=40; *p*	Characterization of ST-EPN with(out) *RELA* and *YAP* fusions or SE-features; Ependymal/subependymal mixed tumors showed a favorable prognosis
Ritzmann et al. (2020)	ST, PF	*n*=302; *p*	No significant differences in PFS rates between ST and PF, most relapses occurred within the first 5 years, late relapses, however, did occur. Chromosome 1q gain and PF-EPN-A were indicators for relapse. Current therapy regimes (resection+ irradiation) insufficiently prolonged survival after relapse
Zschernack et al. (2021)	ST	*n*=18; *p*	Characterization of ST-EPN without *RELA* and *YAP* fusions with *RELA*- like or tanycytic phenotype and alternative fusion-transcripts

**Table 2:** Published pediatric ependymoma patient cohorts enrolled in clinical trials (review of the literature)

Study		No. of patients (*n*)	EFS/OS	Prognostic factors	Implications
Grill et al. (2001)	Multi-center	*n*=73 (<5 years)	4-year EFS/OS 22%/59%	OS: EOR, St tumors	Chemotherapy only or to delay radiation therapy may be suitable for a subset of tumors; however, results were not competitive when compared to other studies
Timmermann et al. (2005)	Multi-center	*n*=34 (<3 years)	3-year EFS/OS 27.3%/55.9%	EFS: EOR	Radiation therapy (in individual cases including the neuroaxis) should be administered
Merchant et al. (2009)	Single center	*n*=153	5-year EFS/OS 74%/85.0%	EFS: gender, age, EOR OS: EOR, WHO grade, ethnic group	Maximal safe surgery and high dose (54–59.4Gy) at an early age (12 months) achieved good results with low risk for 2nd malignancies and brainstem necrosis; age and sex may be used as risk stratification in future trials
Upadhyaya et al. (2019)	Multi-center	*n*=54 (<3 years)	4-year EFS/OS 75.1%/92.6%	EFS: EOR (incl. re-resection), PFA + chromosome 1q gain OS: -	Radiation therapy may be feasible in young children (54Gy)
Merchant et al. (2019)	Multi-center	*n*=356	5-year EFS/OS 62.7%/83.8%	EFS: EOR, chromosome 1q gain in PF-EPN, WHO grade, Gender	Some supratentorial tumors may be cured by complete resection and observation alone, early postoperative radiation therapy (54–59.4Gy) is beneficial, also for patients younger than 3 years
Massimino et al. (2016 + 2020)	Multi-center	*n*=160	5-year EFS/OS 65.4%/81.1% 10-year EFS/OS 58%/73%	EFS: EOR (incl. re-resection), WHO grade, gender; PF-EPN-A, *CDKN2A* deletion, chromosome 1q gain OS: EOR, WHO grade, VP-shunt, gender, age, PF-EPN-A, *CDKN2A* deletion, chromosome 1q gain chromosome 1q gain and *CDKN2A* deletion may be more frequent in children >3 years and are associated with a higher risk of dissemination	Re-resection is warranted, in case of residual tumor a boost of 8Gy (additional to 59.4Gy) may be beneficial and feasible, future trials should include molecular classifications for risk stratification

## Distinction between pediatric and adult ependymoma

Approximately 30% of all ependymomas affect children and adolescent patients [1, 2]. With several differences between pediatric and adult ependymoma, a differentiated approach is warranted. Regarding clinical parameters, intracranial location is frequent in children, while spinal tumors are rather rare compared to the adult population, where this location represents the most frequent manifestation site [1, 2]. Regarding histological classification, anaplastic ependymoma is more frequent in pediatric patients, while myxopapillary ependymomas are mostly found in the adult population [1]; subependymomas are extremely rare in childhood and will therefore not be discussed in this review.

Regarding treatment, complete resection and radiation therapy are administered in a higher frequency in pediatric patients [1]; however, some bias may be owed to the fact that most controlled studies for ependymoma are exclusively including pediatric patients.

With respect to genetic differences, infants with posterior fossa tumors more frequently seem to have balanced genetic profiles compared to polyploid ones in older patients [7, 15]. Furthermore, the gain of chromosome 1q seems to be only slightly more frequent in the pediatric population [16], while PF-EPN-A seem to be merely exclusive to the pediatric population [17]. *RELA*-fusion as well as *CDKN2A* deletion may be detected in pediatric as well as adult patients; however, differences in frequency have not been analyzed in detail so far [9, 11, 12, 18–20].

## Supratentorial ependymoma

Supratentorial ependymomas are more frequently diagnosed in young patients and show a decreasing incidence with age [1].

On MRI supratentorial ependymomas appear as large, inhomogeneously contrast-enhancing tumors with frequent cystic areas as well as calcifications and more rarely hemorrhage and necrosis [21]. Supratentorial ependymomas of childhood are considered to have a better prognosis than those located in the posterior fossa, possibly because gross-total resection is more easily achieved in the former group. Supratentorial ependymal tumors can be divided into three epigenetic groups: ST-EPN-RELA, ST-EPN-YAP, and ST-SE, with supratentorial subependymoma being irrelevant in pediatric patients [11]. Furthermore, there is evidence of supratentorial ependymal tumors different from ST-EPN-RELA nor ST-EPN-YAP [22–24].

### Supratentorial ependymoma with *C11orf95–RELA* fusions

Approximately 70 % of supratentorial ependymomas harbor one of several alternative *C11orf95*–*RELA* fusions resulting from chromosomal instability/microchromothripsis involving the chromosomal region 11q13.1 [11, 12, 20]. *C11orf95*–*RELA* fusion proteins translocate into the nucleus, activating the canonical NF-κB signaling pathway. This pathway controls cell proliferation, apoptosis, and vascularization. In addition, it is involved in inflammatory response [12, 20]. *C11orf95*–*RELA* fusion-positive ependymomas have a densely capillarized phenotype [20, 25], presumably due to the vasogenic action of the NF-κB signaling pathway, as well as a clear cell morphology [26]. Regardless of the specific fusion gene, elevated nuclear protein expression of RelA protein can be identified by immunohistochemistry, offering an accessible cost-effective tool for detection [20, 27]. Furthermore, compared to fusion detection by RT-PCR, immunohistochemistry can identify all different fusions at once using antibodies against p65/RELA (with higher specificity compared to L1CAM) [27], while RT-PCR-based analysis requires specific primers for each individual fusion transcript.

Regarding prognosis, lower survival rates for ST-EPN-RELA ependymoma compared to other ependymal tumors in this location were reported in a retrospective analysis, including adult and pediatric patients [11]. However, these findings are challenged by data derived from exclusively pediatric cohorts enrolled in three recent prospective trials, showing no prognostic disadvantage for ST_EPN-RELA when compared to other ependymoma subtypes [28–30]. In the revised WHO classification of CNS tumors of 2016, supratentorial ependymomas with *C11orf95*–*RELA* fusions were introduced as a novel specific tumor entity [31], initiating the era of a genetically based ependymoma classification.

### Supratentorial ependymoma with *YAP1-MAMLD1* fusion

A small subset of pediatric supratentorial ependymomas harbor a *YAP1-MAMLD1* fusion. This fusion almost exclusively occurs in young children and shows an excellent prognosis, possibly even without further therapy after resection [6, 11, 12, 30]. The involved fusion partners *YES*-associated protein 1 (Yap1) and mastermind-like domain containing 1 (Mamld1) are involved in several pathways, e.g., Hippo signaling pathway and Wnt/β-catenin pathway, active in various cancers [6].

### *CDKN2A* deletion in supratentorial ependymoma

Besides the two aforementioned gene fusions, a frequently reported genetic alteration associated with poorer prognosis in (pediatric) supratentorial ependymomas is homozygous deletion of *CDKN2A* [9, 11, 18, 19]*.* The *CDKN2A* locus on chromosome 9p21 encodes the tumor suppressor proteins p14^ARF^ and p16^INK4A^, which control the cell cycle via Mdm2/p53 or CDK4/6 and the retinoblastoma (RB) family of proteins [32]. Consequently, a deletion/inactivation of *CDKN2A* may result in uncontrolled cell growth. However, only a limited number of cases have been reported so far, and therefore general conclusions should be drawn with caution. In childhood ependymomas, *CDKN2A* deletion is restricted to ependymomas with *RELA* fusion [19].

### Supratentorial ependymoma without* C11orf95–RELA* or *YAP1-MAMLD1* fusions (non-RELA/non-YAP ependymomas)

Finally, supratentorial ependymomas harboring neither *C11orf95*–*RELA* nor *YAP1-MAMLD1* fusions have been reported. Some of these tumors may represent histological mimics of ependymoma misdiagnosed as ependymomas. However, the remaining ones may represent truly novel entities and need to be characterized in more detail [22–24]. These tumors may be sub-stratified, according to their predominant histological appearance and biology as RELA-like, tanycytic, and astroblastoma-like variants. Novel fusion transcripts may be encountered among these tumors as well [24].

## Posterior fossa ependymoma

Pediatric ependymomas are frequently located in the posterior fossa [1, 2]. In addition to histological grading distinguishing between classic (WHO grade II) and anaplastic ependymomas (WHO grade III), there is an association between midline and lateral tumor localization and prognosis.

On MRI, posterior fossa ependymoma may appear as homogenous and well-demarcated tumors with hemorrhage and possible calcification spots showing variable contrast enhancement due to necrosis and cyst formation. Tumors may be located inside the fourth ventricle with possible lateral expansion through the foramina of Luschka or the foramen of Magendie.

With GTR being the strongest predictor of outcome [8, 29, 33–35], tumors arising from the floor and the lateral aspect of the fourth ventricle have a worse prognosis than those arising from the roof. One explanation is that achieving GTR without postoperative deficits is more difficult [36] in the former group. Another explanation may be that the location is associated with different (epi-)genetically driven growth patterns [14, 37].

Analysis of genomic copy number profiles revealed distinct cytogenetic patterns with different prognostic impact. Tumors displaying partial chromosomal alterations (structural alterations) had the worst prognosis, especially those harboring a gain of the q-arm of chromosome 1 [7–9, 33]. Tumors with a balanced genetic profile without any chromosomal alterations were associated with a slightly better prognosis and occur predominantly in very young children [7, 15, 16]. In contrast, whole chromosomal alterations (numerical genetic profile) were found in tumors of older patients and adult—mostly spinal—tumors and associated with a better prognosis [7, 16].

Epigenetically, ependymomas of the posterior fossa can be divided into two defined groups [10, 11, 14], which are discussed in detail in the following paragraphs.

### PF-A ependymoma

PF-A ependymomas occur predominantly in younger children with a decreasing frequency in adolescence and near absence in adulthood. There is a male predominance, and male sex is associated with poorer prognosis [11, 14, 37, 38]. PF-A tumors show a predominantly balanced genetic profile without frequent recurrent somatic mutations, but gain of the long arm of chromosome 1 may be noted in 17–25 % [9, 11, 14, 33, 37]. However, in infants below the age of 18 months, chromosomal aberrations may be absent [15]. Furthermore, CpG-island hypermethylation (compared to PF-B tumors) is present in PF-A tumors [10, 11, 14], caused by overexpression of *EZHIP* (*CXorf67*; enhancer of Zeste inhibitory protein) [37]. By binding EZH2, EZHIP silences targets of the polycomb repressive complex 2 which represses the expression of differentiation genes through trimethylation of H3K27 [10]. Indeed, reduced or absent H3K27me3 expression is characteristic of PF-A ependymomas [39, 40]. The loss of trimethylation of H3K27 can be evaluated by immunohistochemistry, which offers a cost-effective and readily accessible tool to characterize posterior fossa ependymomas. Eventually, DNA methylation analysis revealed further possible subdivision of posterior fossa ependymomas [37, 41]. Pajtler et al. published a subdivision of PF-A ependymomas into 2 main and 9 epigenetically defined subgroups, characterized by different frequencies of H3K27M mutations, gain of chromosome 1q, and levels of CXorf67 or OTX2 expression [37].

### PF-B ependymoma

Compared to PF-A, PF-B tumors are more frequently found in older children and adult patients and frequently show relatively hypomethylated genomes and a polyploid chromosomal profile with gains of complete chromosomes, retained H3K27me3, and slight female predominance [10, 11, 14, 17, 37–41]. Cavalli et al. were able to identify five different epigenetically defined PF-B subgroups distinct from subependymomas, PF-A, and spinal ependymomas [41]. The relatively favorable prognosis of these tumors constitutes a rationale for reduction of adjuvant therapy after surgery for these tumors, avoiding the long-term sequelae of radiotherapy.

## Spinal ependymoma

In contrast to the adult population, spinal ependymomas are rare in children [1, 2]. Furthermore, pediatric patients suffering from ependymomas in this location seem to be generally older than those with supratentorial or infratentorial tumors [1, 2]. Spinal ependymomas can be histologically classified into myxopapillary ependymoma WHO grade I, as well as classic (WHO grade II) and the less frequent anaplastic ependymoma (WHO grade III) [31, 42, 43]. Additionally, a rare and aggressive genetic variant with *MYCN* amplification has been described [44–46].

### Classic and anaplastic spinal ependymoma

Classic and anaplastic ependymomas arise predominantly in the cervicothoracic cord and are located intramedullary [42]. On MRI, they appear as T1-hypo- and T2-hyperintense intramedullary, contrast-enhancing lesions with frequent cystic, hemorrhagic, and necrotic components as well as possible calcifications. About 60 % are associated with syringomyelia [47]. Classic and anaplastic spinal ependymomas have a predominantly numerical cytogenetic profile, similar to PF-B ependymomas and are associated with a better prognosis [7, 16]. The most frequent chromosomal alteration in spinal ependymomas is the loss of the long arm of chromosome 22 [11], and the most frequent somatically mutated gene in classic and anaplastic spinal ependymoma is *NF2,* located on chromosome 22q [48], while *NF2* is neither mutated in intracranial nor myxopapillary ependymomas*.* Epigenetically, based on a mixed and predominantly adult cohort, spinal ependymomas were subdivided into three groups SP-SE, SP-EPN, and SP-MPE [11]. Spinal ependymomas show a methylation signature distinct from subependymomas, myxopapillary ependymomas, and *MYCN*-amplified (anaplastic) ependymomas [11, 46] (see below). Overall, patients with spinal ependymomas have a favorable prognosis regardless of age with progression-free and overall survival rates of 70–90 and 90–100 %, respectively [49] as well as limited evidence of a worse prognosis for anaplastic tumors.

### Myxopapillary ependymoma

The name is derived from their histopathological features which include perivascular “sleeves” of myxoid ground substance, microcysts, and radial (pseudopapillary) arrangements of the tumor cells around vessels. In contrast to classic and anaplastic tumors, myxopapillary ependymomas are mostly found in the conus-cauda region in intradural but extramedullary location [42, 50] and are relatively rare in pediatric patients [51]. Multifocal growth as well as locations other than the conus-cauda region [52, 53] and extra spinal manifestation in the sacrococcygeal soft tissue [54] have been described. Furthermore, with a given tendency to leptomeningeal dissemination, craniospinal MRI is warranted [55].

The diagnosis of myxopapillary ependymoma is straightforward if typical histological features are present. However, even if these are absent, their distinct methylation profile will establish the diagnosis, since it differentiates them from classic/anaplastic ependymomas [11, 17, 26]. Of note, rare anaplastic variants with an increased mitotic index as well as vascular proliferation and necrosis have been described [54]; these features do not appear to influence survival, though [52]. Overall survival of myxopapillary ependymoma is favorable with >90 % at 10 years [51, 56, 57]. Progression-free survival is less favorable, with frequent persistent and recurrent disease as well as dissemination sometimes already present at diagnosis [56, 57]. In incompletely resected cases, adjuvant radiotherapy improves the prognosis [58].

### Spinal ependymoma with *MYCN* amplification

A rare and highly aggressive subtype of anaplastic spinal cord ependymoma with *MYCN* amplification predominantly affecting young adults but older children as well has been identified recently, further supporting the prognostic relevance of biological classification. Their site of predilection is the cervico-thoracic spine [44–46, 59], and tumors are often large at diagnosis, involving multiple spinal segments and displaying an exophytic growth pattern as well as frequent dissemination [44, 46, 59]. Histologically, ependymomas with *MYCN* amplification present with characteristic ependymal features and signs of anaplasia [44, 46, 59]. *MYCN* protein overexpression can be demonstrated by immunohistochemistry [44, 59]. Genetically, all cases show high-level *MYCN* amplification, which remains stable throughout the course of disease [44–46, 59]. This subtype also shows a distinct methylation profile [44, 59]. The cases reported so far showed unfavorable progression-free and overall survival. Therefore, their distinction from other spinal ependymomas is essential.

## Treatment strategies according to the results of latest trial cohorts

### Published trial cohorts

The most important factor in the treatment of pediatric ependymoma, regardless of age and location, is maximal safe surgery [28–30, 33–35, 60, 61], ideally GTR, and may include second surgery, whenever needed and considered feasible [28, 30, 34]. The mainstay of approved adjuvant treatment has been postoperative radiation therapy regardless of location and extend of resection [34, 35]. Weighing possible long-term sequelae against tumor recurrence, the cut-off age for radiation therapy is currently debated; it is performed as early as 12 months in recently published studies [29, 30, 34, 35]. The most favorable single center results so far reported for a cohort of pediatric ependymoma with supra- and infratentorial location were achieved by Merchant and colleagues [35] published in 2009 with 5-year EFS and OS of 74 % and 85.0 %, respectively, while promising multi-center results were published by Massimino et al. in 2016 [34] with 5-year EFS and OS of 65.4 % and 81.1 % and consecutive 10-year EFS and OS rates of 58 % and 73 % [28, 34], respectively; the largest cohort published to date (*n*=356) also achieved good 5-year EFS and OS rates of 62.7 % and 83.8 %, respectively [29]. Since local recurrence is reported more frequently than distant failure [34, 62, 63], Massimino et al. advocated local intensification of RT with an extra boost on the tumor region [34].

### Genetic parameters, prognostic, and possible therapeutic implications

Regarding the different emerging biologically defined subtypes, Merchant et al. recommended an observation-only approach for completely resected WHO grade II supratentorial tumors with promising results [29]. A similar approach for PF-B-type tumors was suggested by Ramaswamy et al. [38]. The option of observation only for certain subentities is currently investigated in the SIOP-Ependymoma II trial and in the ACNS0831 trial. In contrast to PF-B tumors, PF-A-type ependymoma with gain of the q-arm of chromosome 1 showed a dismal prognosis and needed maximal safe surgery as well as radiation- and chemotherapy, especially since local and distant failure and the tendency for dissemination are more frequently reported compared to other ependymoma subtypes [28, 29]. For supratentorial ependymoma, the negative prognostic impact of *RELA*-fusion-positive ependymoma that was initially reported [11] could not be validated by others [29, 30]. On the other hand, recent data by Jünger et al. and Massimino et al. suggest that supratentorial ependymoma with *CDKN2A* deletion represents a more aggressive variant, possibly requiring intensified treatment [19, 28]. Given Palbociclib, an oral inhibitor of *CDK4/CDK6*, is currently being tested for clinical efficacy in high-grade gliomas with amplification of *CDK4/CDK6* or homozygous deletion of *CDKN2A* [64]*, CDKN2A* deletion in pediatric supratentorial ependymomas should be the subject of further investigation since it might represent a therapeutic target. Based on the advancing characterization of the biological features of ependymoma, targeted therapies may be identified and proven in a clinical setting in the future [65]. However, for now, maximal safe surgery remains the upfront goal and postoperative radiation therapy the most effective treatment. Furthermore, despite ependymoma being divided into biologically distinct sub-entities, WHO grading was used for risk stratification in most trials and confirmed to be a significant predictor of prognosis [28, 29, 34, 35]. Gender and age might be prognostically relevant as well [29, 34, 35]. However, the prognostic impact of age may be due to the fact that all treatment modalities are not necessarily used for very young children in all departments.

### Radio- and chemotherapy

Regarding proton versus photon therapy, the two modalities are considered of equal efficacy. Regarding sequelae, proton beam therapy, however, may be more suitable for ependymoma typically arising at very young age in order to spare the highly vulnerable surrounding developing brain [62, 63]. For clinical validation, further evaluation in prospective trial cohorts with longer follow-up is needed.

To date, chemotherapy according to different protocols was often administered in children with incompletely resected or anaplastic (WHO grade III) tumors in addition to radiotherapy [29, 30, 34]. In very young children, chemotherapy was applied with the aim to delay radiation therapy. However, efficacy of chemotherapy could not be proven [60, 61]. The value of chemotherapy in ependymoma therapy is currently being re-evaluated in the ongoing SIOP Ependymoma II trial. Furthermore, the ACNS 0831 trial, also investigating the possible benefit of chemotherapy, has shown first promising results, indicating a possible benefit of maintenance chemotherapy regarding EFS (presented at ISPNO 2020).

### Therapy of spinal ependymoma

Data on treatment protocols specific for pediatric patients with spinal ependymomas are sparse, since pediatric trials focused on intracranial ependymoma and the majority of larger retrospective analyses described mixed or adult cohorts. Data from the prospective HIT-trial on 29 spinal ependymomas have shown gross-total resection as the main predictor of favorable outcome [49]. However, uniform adjuvant treatment protocols for pediatric spinal ependymomas do not exist [49] and the relevance of postoperative radio- and chemotherapy is not proven. Nevertheless, the European Association of Neuro-Oncology (EANO) recommended postoperative radiotherapy for all anaplastic (WHO grade III) tumors and incompletely resected classic (WHO grade II) tumors [55]. For myxopapillary ependymomas, radiotherapy was recommended even after complete resection as well as in relapsed cases after—if feasible—second surgery in combination with chemotherapy [55]. For myxopapillary ependymomas which have a higher rate of local recurrence than classic grade II tumors, GTR seems to be the only predictor of progression-free and overall survival. Relapse rates for pediatric patients seem to be generally higher when compared to adult patients [66].

### Treatment of primarily metastatic ependymoma and recurrent ependymoma

CNS metastases in ependymoma are rare at presentation [31]. Benesch et al. recently published data of ten such pediatric patients (2.4 % of 402) enrolled in the multi-center HIT-2000 ependymoma trial [67]. The four patients with ST-EPN-RELA were alive in first or second remission 7.5–12.3 years after initial diagnosis, while all 4 with PF-EPN-A had died, indicating that chemotherapy may be required for these patients and that genetic subgrouping may adequately predict prognosis [67]. With regard to radiotherapy, in disseminated ependymomas, the full craniospinal volume will be targeted by the radiotherapy, whereas in localized tumor, only the treatment of tumor bed is standard of care [55].

For recurrent ependymomas, the EANO suggests reoperation and reirradiation if possible as well as chemotherapy. If no local treatment is feasible, chemotherapy can be administered alone [55]. However, prognosis of children with recurrent ependymoma remains poor [68]. A recently published retrospective analysis reported an increased risk for recurrence in patients with tumors with gain of chromosome 1q and/or PFA-methylation profile, further demonstrating only little benefit from resurgery and reirradiation in case of relapse [69].

## Conclusion

In conclusion, pediatric ependymomas comprise biologically distinct tumor entities with different (epi)genetics, age distribution and localization, as well as different prognosis. However, histological classification within these biologically defined entities is still relevant for risk stratification. Furthermore, molecular classification gains importance and will be addressed in the upcoming WHO classification of CNS tumors.

To date the mainstay of treatment remains maximal safe surgery (ideally GTR), achieved with intraoperative monitoring and neuronavigation and if necessary second surgery. Adjuvant radiation therapy as soon as justifiable, given potential harm to the surrounding nervous tissue, is recommended for most tumors; however, there is substantial evidence that it might be spared in defined subentities, while the role of chemotherapy still needs to be established.

### Remaining open questions

Since pediatric ependymomas depict rare entities, all children should be treated in clinical trials, analyzing demographic, clinical, histological, molecular, and treatment-derived parameter in order to answer the questions remaining to date:

i) Is a mere molecular risk stratification superior to classic histopathological classification to differentiate between low- and high-risk tumors?
ii) Does surgery alone depict sufficient treatment for low-risk tumors?
iii) What time, dose, and extent are suitable for which type of tumor in terms of photon and proton radiation; and which modality may be more suitable?
iv) What is the role of classic chemotherapy in pediatric ependymoma?
v) With potential therapeutic targets identified, what will be the role of targeted therapy?
vi) Which is the optimal treatment for recurrent and primarily metastatic pediatric ependymoma; do we need a more aggressive treatment regime?
